# Higher pre-infection vitamin E levels are associated with higher mortality in HIV-1-infected Kenyan women: a prospective study

**DOI:** 10.1186/1471-2334-7-63

**Published:** 2007-06-26

**Authors:** Susan M Graham, Jared M Baeten, Barbra A Richardson, Daniel D Bankson, Ludo Lavreys, Jeckoniah O Ndinya-Achola, Kishorchandra Mandaliya, Julie Overbaugh, R Scott McClelland

**Affiliations:** 1 Department of Medicine, Box 356420, University of Washington, Seattle, Washington 98195, USA; 2 Department of Biostatistics, Box 357232, University of Washington, Seattle, Washington 98195, USA; 3 Department of Laboratory Medicine, Box 357110, University of Washington, Seattle, Washington 98195, USA; 4 Department of Epidemiology, Box 357236, University of Washington, Seattle, Washington 98195, USA; 5 Department of Pathology and Laboratory Medicine Service, Veterans Affairs Puget Sound Health Care System, 1660 South Columbian Way, Seattle, Washington 98108, USA; 6 Department of Medical Microbiology, University of Nairobi, P.O. Box 30197-00100, Nairobi, Kenya; 7 Coast Provincial General Hospital, P.O. Box 90231, Mombasa, Kenya; 8 Division of Human Biology, Fred Hutchinson Cancer Research Center, P.O. Box 19024, Seattle, Washington 98109, USA

## Abstract

**Background:**

Low vitamin E levels are often found in HIV-1 infection, and studies have suggested that higher levels may decrease the risk of disease progression. However, vitamin E supplementation has also been reported to increase CCR5 expression, which could increase HIV-1 replication. We hypothesized that vitamin E levels at HIV-1 acquisition may influence disease progression.

**Methods:**

Vitamin E status was measured in stored samples from the last pre-infection visit for 67 Kenyan women with reliably estimated dates of HIV-1 acquisition. Regression analyses were used to estimate associations between pre-infection vitamin E and plasma viral load, time to CD4 count <200 cells/μL, and mortality.

**Results:**

After controlling for potential confounding factors, each 1 mg/L increase in pre-infection vitamin E was associated with 0.08 log_10 _copies/mL (95% CI -0.01 to +0.17) higher set point viral load and 1.58-fold higher risk of mortality (95% CI 1.15–2.16). The association between higher pre-infection vitamin E and mortality persisted after adjustment for set point viral load (HR 1.55, 95% CI 1.13–2.13).

**Conclusion:**

Higher pre-infection vitamin E levels were associated with increased mortality. Further research is needed to elucidate the role vitamin E plays in HIV-1 pathogenesis.

## Background

Vitamin E plays a complex role in maintaining immune function. Low levels of this micronutrient have been found in HIV-1-infected individuals with both asymptomatic and advanced disease [1,2]. *In vitro*, vitamin E has been reported to inhibit HIV-1 transcription and to suppress the activation of virus in latently infected cells [3,4]. Generally, a protective effect in HIV-1 infected persons has been assumed [5]. However, the effect of vitamin E on HIV-1 disease progression has not been well characterized, and its overall role remains unclear. Moreover, recent evidence suggests that vitamin E supplementation may increase CCR5 expression, which in turn could increase HIV-1 replication [6].

In prospective studies of HIV-1-infected people, higher vitamin E levels and higher intake have been associated with a lower risk of progression to AIDS [7,8]. However, vitamin E levels can decrease as HIV-1 infection progresses, and deficiencies found late in disease may result from advanced illness [9]. Most studies have been limited in their ability to determine the temporal sequence of HIV-1 acquisition and development of deficiencies. In addition, studies showing an advantage to high levels of vitamin E may have been confounded by factors (e.g., socioeconomic status) associated both with better nutritional intake or supplement use and with better HIV-1 disease outcomes.

Prospective studies of individuals who have been followed since prior to HIV-1 seroconversion provide a unique opportunity to study the effect of nutritional status before acquisition of the virus on the progression of HIV-1 disease. The Mombasa cohort was established to evaluate risk factors for HIV-1 acquisition and disease progression in a population of female sex workers. The purpose of this analysis of prospectively collected data from the cohort was to test the hypothesis that pre-infection vitamin E levels would be associated with HIV-1 disease outcomes.

## Methods

Beginning in February 1993, HIV-1-seronegative female sex workers attending a municipal clinic in Mombasa, Kenya were invited to participate in a prospective cohort study of risk factors for HIV-1 acquisition. Women were interviewed at study enrollment and at monthly intervals thereafter. A physical examination performed at each visit included specimen collection for diagnosis of sexually transmitted diseases. In addition, a blood sample was obtained for HIV-1 serologic testing. Women who seroconverted to HIV-1 during follow-up were asked to continue monthly clinic visits, and quarterly CD4 counts were obtained beginning in April 1998.

Services provided to study participants included HIV-1 counseling and testing, diagnosis and treatment of sexually transmitted diseases, and general outpatient care. For HIV-1 infected women, care included trimethoprim-sulfamethoxazole prophylaxis and treatment of opportunistic infections. During the study period, no women reported receiving antiretroviral therapy, which had limited availability in Kenya before 2004. Starting in March 2004, free antiretroviral therapy was provided to cohort participants according to WHO and Kenyan Ministry of Health Guidelines.

Participants who missed two consecutive clinic appointments were traced by study staff. For women who died, information on the date of death was collected from family, employers, or colleagues during tracing visits. From 1993 to 2000, we obtained verbal informed consent after participants either read or listened to a study nurse read a standardized consent document. Beginning in 2000, we implemented written consent for all new and continuing participants. This research was approved by the University of Washington, University of Nairobi, and Fred Hutchinson Cancer Research Center ethical review committees.

HIV-1 serologic testing was performed by an ELISA (Detect HIV, BioChem ImmunoSystems, Montreal, QC, Canada) and positive results confirmed by a second ELISA (Recombingen, Cambridge BioTechnology Ltd., Worcester, MA, USA). For women who seroconverted during follow-up, plasma samples from the two clinic visits before seroconversion were tested for HIV-1 RNA, using the Gen-Probe HIV-1 viral load assay [10,11]. A level of ≥100 copies/mL was used to define detection of virus in plasma. When RNA was detected, the date of infection was estimated at 17 days before the first RNA-positive sample. For women who had no plasma viremia detected before HIV-1 seroconversion, the date of infection was estimated at the midpoint between the last seronegative and the first seropositive visit. Women with >1 year between these two visits were excluded unless a positive HIV-1 RNA level was present before seroconversion, because the date of infection could not be estimated with sufficient precision.

Absolute CD4 counts were measured by one of two methods (Cytosphere, Beckman Coulter Corporation, Miami, FL, USA, or Zymmune, Bartels, Inc., Issaquah, WA, USA), which have been demonstrated to generate comparable results [12]. The first plasma viral load between 4 and 24 months after HIV-1 infection was taken to be the set point viral load.

Vitamin E levels in plasma were assessed by high performance liquid chromatography [13]. For 7 participants, a serum sample was the only pre-infection sample available. Because values measured in serum and plasma are comparable within the normal range [14], stored serum or plasma samples were used for assessment of vitamin E.

SPSS (version 12.0; SPSS Inc., Chicago, IL, USA) was used for data analysis. The viral load was set at 50 copies/mL for 2 samples with an HIV-1 RNA set point <100 copies/mL. Linear regression was used to estimate associations between pre-infection vitamin E level and set point viral load. Cox proportional hazards regression analysis was performed to evaluate the association between pre-infection vitamin E and time to CD4 count <200 cells/μL or death. Follow-up was censored at death or the last clinic visit. Multivariate models were used to control for potential confounding factors of the association between vitamin E status and HIV-1 disease progression, including hormonal contraceptive use and genital ulcer disease at the time of infection [11].

## Results

Between February 1993 and August 2001, 228 women seroconverted for HIV-1. Data on the follow-up of these women through March 2004 were included in this analysis. Sixty-seven (29%) were excluded because the date of infection could not be estimated with sufficient precision. Ninety-four women (42%) did not have blood samples available from the time points of interest (i.e., pre-infection and set point samples). The remaining 67 (29%) were eligible for analysis in this study, and vitamin E was assessed at the last pre-seroconversion visit at which no HIV-1 RNA was detected.

Pre-infection samples were collected a median of 30 days (IQR, 16 – 59) before HIV-1 infection. The median pre-infection vitamin E level was in the low-normal range (7.5 mg/L; IQR, 5.2 – 9.5) and the prevalence of vitamin E deficiency (<5 mg/L) was 21%. Baseline characteristics for the 67 women included are summarized in Table 1. Women included in the analysis had a lower number of sex partners and sexual frequency in the 6 months preceding HIV-1 infection, but did not differ significantly from the 161 seroconverters excluded from this study for any of the other characteristics presented in Table 1.

**Table 1:** Characteristics of 67 Kenyan women at the time of HIV-1 infection

Characteristic	N (%) or Median (IQR)
Age, years	28 (25 – 35)
Education, years	8 (7 – 10)
Live births	1 (1 – 2)
Sex partners per week*	1.0 (0.7 – 1.0)
Sex frequency per week*	1.3 (1.0 – 2.0)
Frequency of condom use (%)*	83 (40 – 100)
Use of hormonal contraceptive method	
Oral contraceptive pill	15 (22.4%)
DMPA†	23 (34.3%)
Norplant	1 (1.5%)
None reported	28 (41.8%)
Genital ulcer disease	3 (4.5%)
Time in cohort before infection, months	9 (3 – 21)

* Average values per week for each woman, for the 6 months preceding HIV-1 infection.

† Depot medroxyprogesterone acetate

The 67 women were followed for a median of 68 months (IQR, 45 – 85). Set point samples were collected a median of 199 days (IQR, 154 – 267) after HIV-1 infection. The median set point viral load was 4.7 log_10 _copies/mL (IQR, 3.9 – 5.2). CD4 lymphocyte counts were available for 55 women (82%). Nineteen women (28%) progressed to a CD4 count <200 cells/μL, and twelve women (18%) died during follow-up: 1 with pneumonia, 1 with fever, 1 with hematemesis and abdominal distention, and 9 of unknown causes. Each 1-unit increase in log_10 _set point viral load was associated with a hazard ratio of 1.31 (95% CI, 0.83–2.07, p = 0.248) for the risk for progression to a CD4 count <200 cells/μl and 1.66 (95% CI, 0.80–3.45, p = 0.176) for the risk of death.

After adjustment for potential confounding factors, each 1 mg/L increase in pre-infection vitamin E level was associated with a 0.08 log_10 _copies/mL higher set point viral load (Table 2). No significant association was found between pre-infection vitamin E and time to CD4 count <200 cells/μL. Higher pre-infection vitamin E levels were associated with a 1.16-fold higher mortality risk. After adjustment, this association was strengthened to a 1.58-fold increase in mortality risk. Higher mortality persisted after further adjustment for set point viral load (adjusted hazard ratio 1.55, 95% CI 1.13 – 2.13, p = 0.006).

**Table 2:** Association between pre-infection vitamin E status and measures of HIV-1 disease progression

Outcomes	β (95% CI) or HR (95% CI)	*P*	Adjusted* β (95% CI) or HR (95%CI)	*P*
Set point viral load (log_10 _copies/ml)	+.09 (+.01, +.17)	0.038	+.08 (-.01, +.17)	0.066
CD4 count <200 cells/μL	0.99 (0.86, 1.13)	0.85	1.02 (0.88, 1.18)	0.79
Mortality	1.16 (1.02, 1.33)	0.024	1.58 (1.15, 2.16)	0.004

β, mean change in outcome for each 1 mg/L increase in vitamin E (linear regression); CI, confidence interval; HR, hazard ratio (Cox proportional hazards regression).

* Adjusted for age at infection, education, parity, hormonal contraception at infection, GUD at infection [11].

## Discussion

In this study of women with well-defined dates of HIV-1 infection, vitamin E levels before HIV-1 infection were associated with higher mortality. For each 1 mg/L increase in pre-infection vitamin E, there was an approximately 60% higher relative risk of death. An association between higher vitamin E levels and higher set point viral load was of borderline significance. However, the higher mortality risk persisted after adjustment for set point viral load, suggesting that the association between pre-infection vitamin E and mortality may be independent of its effect on the plasma viral load at set point.

A higher prevalence of vitamin E deficiency in HIV-1-infected individuals than in uninfected controls was reported soon after HIV-1 testing became available [1]. Few prospective studies of nutritional status in HIV-1-infected persons have specifically analyzed vitamin E levels. In a Baltimore cohort, HIV-1-seropositive men in the highest quartile of vitamin E levels at enrollment showed a lower risk of progression to AIDS compared with the rest of the cohort (HR 0.67, 95% CI 0.45–0.98), although there was no apparent relationship between low vitamin E levels and risk of progression to AIDS or mortality [7]. In a Miami cohort of HIV-1-seropositive women, vitamin E status was not significantly associated with survival [15]. Thus, evidence for a possible association between vitamin E levels and HIV-1 disease progression is not conclusive.

The mechanism by which higher vitamin E could accelerate HIV-1 disease progression is unknown, but vitamin E has been found to influence the production of RANTES, an important antiviral chemokine. In a small supplementation study, 500 mg of alpha-tocopherol (the most potent of the tocopherols with vitamin E activity) given once daily for 2 months reduced RANTES production and increased the expression of CCR5 on the surface of CD4+ T cells of HIV-1 infected persons [6]. Endogenous production of antiviral factors like RANTES may significantly affect HIV-1 replication and the pace of disease progression [16]. Increased CCR5 expression and decreased RANTES levels would be expected to increase HIV-1 replication.

There are three important strengths of this study. First, because the women were followed prior to seroconversion, vitamin E levels could be tested on samples collected before HIV-1 acquisition, when low vitamin E status could not be a result of disease progression. Second, the frequent follow-up allowed measurement of pre-infection vitamin E status within 2 months of the estimated time of infection. Third, outcomes accrued over several years, and there was active tracing of participants lost to follow-up.

There were some limitations to this study. First, CD4 cell counts were not available before April 1998. Thus, for some women, the first CD4 measurement was <200 cells/μL, decreasing our ability to measure progression to this endpoint with precision. Second, a reliable estimate of the time of infection and the required samples were available for only a minority of seroconverters in the Mombasa cohort (29%). Although it reduced the sample size, this limitation did not compromise the internal validity of the study, and we still found significant associations between vitamin E status and both set point viral load and HIV-1 disease progression. Third, the cause of death was unknown for most of the women included, and not all deaths can be attributed to HIV-1 disease despite rigorous efforts to trace women. However, survival among HIV-1-seroconverters in our cohort prior to antiretroviral therapy is similar to that of other seroconverter cohorts in which HIV-1 has been causally related to death among those infected [17,18]. Fourth, most of the women in our cohort are infected with HIV-1 subtype A, which has been associated with a slower rate of disease progression, compared to other subtypes [19]; this may have influenced the associations we detected.

Finally, no data were collected on vitamin E intake for these women. Vitamin E status is influenced by a number of factors (e.g., dietary fat intake, body mass index, smoking, seasonal variation in food availability), and the vitamin E levels found in this study were similar to those reported previously in studies of African females [20,21]. This study was not designed to test the hypothesis that higher vitamin E intake or vitamin E supplementation would influence HIV-1 disease progression.

## Conclusion

In conclusion, the findings of this study provide evidence that higher vitamin E levels may have undesirable effects in HIV-1 infection. Further research is needed to determine whether these findings can be replicated in other cohorts and to better understand the mechanisms by which vitamin E may influence HIV-1 replication and disease progression.

## Abbreviations

AIDS – Acquired ImmunoDeficiency Syndrome

CCR5 – Chemokine (C-C motif) Receptor 5

ELISA – Enzyme-Linked ImmunoSorbent Assay

HIV – Human Immunodeficiency Virus

RANTES – Regulated on Activation Normal T-Cell Expressed and Secreted

RNA – RiboNucleic Acid

## Competing interests

The authors declare that they have no competing interests.

## Authors' contributions

SMG coordinated the data, performed the statistical analysis, and drafted the manuscript. JMB and BAR provided statistical support. JMB, DDB, and RSM conceived the study. LL, JON, KM, and JO participated in study design and coordination. All authors read and approved the final manuscript.

Presented in part at the 13^th ^Conference on Retroviruses and Opportunistic Infections, Denver, Colorado, 5–9 February 2006 (abstract 290).

## Pre-publication history

The pre-publication history for this paper can be accessed here:


